# Winter School on sEMG Signal Processing: An Initiative to Reduce Educational Gaps and to Promote the Engagement of Physiotherapists and Movement Scientists With Science

**DOI:** 10.3389/fneur.2020.00509

**Published:** 2020-06-24

**Authors:** Carlos De la Fuente, Álvaro S. Machado, Marcos R. Kunzler, Felipe P. Carpes

**Affiliations:** ^1^Carrera de Kinesiología, Departamento de Ciencias De la Salud, Facultad de Medicina, Pontificia Universidad Católica de Chile, Santiago, Chile; ^2^Laboratorio LIBFE, Escuela de Kinesiología, Universidad de los Andes, Santiago, Chile; ^3^Centro de Salud Deportivo, Clínica Santa María, Santiago, Chile; ^4^Laboratory of Neuromechanics, Universidade Federal Do Pampa, Uruguaiana, Brazil

**Keywords:** surface electromyography, education, knowledge transfer, teaching, neurorehabilitation engineering

## Abstract

The application of surface electromyography (sEMG) in neurology is sometimes limited by a scientific background in the use of sEMG. Students frequently use sEMG only when developing their graduate studies. To reduce these barriers, we promoted a free Winter School on sEMG to Latin American students. The school was a 3-day event with theoretical classes and computer programming in Matlab. Lectures were delivered in Portuguese and Spanish to 50 participants. All lectures were recorded and made available on YouTube®. After the School, participants completed a written exam to receive a certificate. The written exam revealed the average effectiveness of 71 ± 20% in the comprehension of topics addressed during the school. Participants rated the School as “excellent” and considered the event as having changed their thoughts about the use of sEMG. Limited mathematical skills or background were the main barriers identified to follow the lectures and to make use of sEMG. We conclude that the Winter School had a positive impact on participant's formation, especially by showing them the importance of continuous involvement with the concepts related to sEMG to become proficient in its use. From the participant's point of view, the activity was excellent and the follow up of the school on YouTube® suggests that combining face-to-face activities followed by the online availability of lectures is a valid strategy to reinforce the learning process and to reduce barriers in the use of sEMG. Whether similar results would be achieved for a paid registration event in an economically developing region, still requires further investigation.

## Introduction

Latin America is one of the most unequal regions in the world based on the Gini Index (1). Therefore, low cost educational and science opportunities are a milestone in providing access to knowledge and social mobility, providing educational innovation with the opportunity to be prepared for the burgeoning of information technologies, globalization, and changes in the knowledge (2). This inequality in knowledge also concerns students and professionals whose professional actuation is often limited by the lack of a scientific background on the theoretical and technical aspects of techniques which can contribute in a relevant way to therapy and assessment in rehabilitation e.g., sEMG (3–5). These barriers to the wide use of techniques like sEMG have different sources. We consider that restriction in universal access to higher education, as observed in economically developing countries, plays a major role in these barriers. Furthermore, in these regions, once a student joins the university, not all institutions will be able to offer research laboratories to complement lectures or to support research groups, limiting the experiences that the student will have during the professional formation. In Brazil, most of the exposition to science happens for students attending free public universities, which are also the institutions producing most of the scientific research in that country and Latin America. However, public universities promoting free education are not available in all countries and sometimes have a bad geographical distribution.

For example, in Chile, which is considered an emergent economy, social inequality remains high, like in other Latin American countries, and education is considered a consumer good. It means that even public education has a high cost, which can be higher than the cost of studying at private institutions. It results in science being transformed to privilege people with better economic conditions and credit capacity, which ends up segregating the society (6). In Brazil, public universities are free for students and in the past 15 years there was a strong effort to promote a better geographical distribution of public universities to benefit a larger number of people, including social programs to help families send young family members to universities. It may help to explain some of the differences between Brazil and other countries in Latin America, regarding scientific research, and development in some fields.

In addition to the economic barriers, there are other barriers requiring attention. These include weak, or absence of a proper educational background to move forward in concepts related to higher education. One important topic of education and research widely used in the fields of physical education, physiotherapy, and engineering is surface electromyography (sEMG). However, many barriers are observed in the use of this tool, as we will further discuss in this article.

sEMG encompasses the measurement of electrical signals involved in the muscle contraction and is quantified using surface electrodes, with applications in humans (7) and other animals (8). Over the past decades, studies on nerve conduction (9) and other applications of sEMG have increased both in number and quality, and a few textbooks have been written on sEMG (10–15). Furthermore, recent technological advances permit the collection of signals in the field using wireless sensors, and hardware has become more accessible at a lower cost because many companies are providing different solutions for data acquisition. However, the same technological development that enabled tablets and smartphones to process and analyze biological signals used in clinical practice and research, making the access to sEMG easier, has created a scenario where programming and analytical thinking sometimes is no longer necessary (16). While facilitating the spread of sEMG use, this reduces the immediate need to understand the calculations involved in data acquisition, processing, and analysis, causing a gap/barrier between theory and practice in students and professionals from the health and life sciences. In this regard, if these topics are not adequately covered during university studies, there is a high chance of increased misuse in professionals with a weak technical background who start to use tools like sEMG.

For example, to promote better use of the sEMG in clinics, hospitals, and gymnasiums, knowledge of several basic concepts from anatomy, neurophysiology, and physics, among others, will be required. The user needs to properly locate the electrodes and be aware of particularities depending for example on the muscle evaluated (17, 18). Similarly, sEMG users must understand the mathematical aspects of signal acquisition and processing, the risks of making mistakes, and the risks of subjective interpretations. For a deeper understanding of these topics, we recommend visiting http:www.robertomerletti.it and www.seniam.org, where a wide number of sources for historical and updated academic material are available to help elementary and advanced sEMG studies.

The real condition is that most health professionals from Latin America do not receive a background in mathematics sufficient enough to understand and use sEMG in their professions. Many students seek training in health courses just to get away from mathematical work. National scientific journals related to human movement sciences are available, and most of the papers published in these journals, addressing sEMG, are the result of laboratory research developed during master's and doctoral programs. These papers are accessible to students, but the use of scientific papers during the study of these topics needs to be strongly promoted. This establishes an important barrier to sEMG applications that have been considered when promoting regular congresses in the field, like the Brazilian Congress of Biomechanics, and the Congress of the Chilean Association for Human Movement Sciences. But this limitation is not observed only in economically developing countries (EDC).

The evaluation of the knowledge from participants working in developed countries also suggested that neurology residents show a knowledge gap regarding the use of sEMG, resulting in up to a quarter of residents expressing a lack of confidence when using techniques like sEMG (19). Furthermore, life sciences students report difficulties in carrying out laboratory calculations (20). In this study, the promotion of educational activities like workshops improved the level of competence in a laboratory environment (20). This lack of confidence, the knowledge gap, and difficulties with laboratory calculations can influence the use of sEMG. As a result, sEMG results provided by automated routines and which does not require a higher level of technical knowledge, could both help and be a trap for users. Software and graphical interfaces using custom made codes are very useful (21), but they do not exclude the importance of the user's knowledge when acquiring and processing the biological signals. We could exemplify this by considering an sEMG examiner making use of a graphical interface that requires a choice of parameters like the criteria for activation on/offset identification (21). Such a tool, like others available for free on the internet, can be a powerful and a valuable instrument in the clinical field, research, and also from an educational perspective, but the user needs basic knowledge to make the correct configurations and to avoid bias in the sEMG analysis (22). The current movement toward open science increases the sharing of codes for example, and sometimes the sEMG user can get a code from a colleague to use on their own data, but the basic knowledge on how to use it remains obligatory so as to avoid misleading results or to generate new incorrect data. Having a code and the skill to run it will, therefore, not be enough to overcome the barriers to proper use of the technique.

The knowledge about sEMG may also impact on aspects related to patient management. When considering the use of sEMG in a clinic or hospital, a patient's education about sEMG is also important. We consider that in some cases, not informing the patient or participant about what the examination is, and how it works, may increase stress and possibly change participants' behavior when realizing that many electrodes are going to be attached to their own body, and this misunderstanding may lead to limited clinical results. Among middle-aged patients undergoing sEMG examination, 52.1% of the patients either received no information about what sEMG is, or, the information provided was very poor or incorrect (23). On the other hand, these data may reflect the lack of knowledge of the sEMG user in charge of the examination who is unable to properly explain what is going to happen. Different groups of scientists try to provide solutions to reduce these effects and companies also try to improve the software for each new version launched.

Considering all these aspects, we promote educational activities that reduce the knowledge gap and barriers faced when using sEMG, observed among health professionals who want to or already do use of sEMG without proper training in basic concepts related to its use. In 2018 we organized the first Winter School on sEMG Digital Signal Processing for Latin American students. Here we describe this educational activity, and the impact it had as perceived by the participants. We also share our experience in the selection of the topics for lectures and the methodology for the development of the course. The barriers faced when using sEMG are also discussed.

## Activity Description

The Winter School lasted for 3 days during September 2018 and was held at the Universidade Federal do Pampa, a Brazilian public university established in a remote region of the Rio Grande do Sul state in Brazil. Registration was free of charge and we received 50 applications for registration. The event was advertised on social media and through email lists of different institutions. There was no limitation in the background level required or the purpose of the participants registering to the school, and the program was available to all participants before completing the free registration. We did not control registration according to the level of knowledge of the participants because it has been suggested that merging students with different levels of expertise may bring education advantages especially for those who are less experienced (24). Upon registration, participants received a customized handout including brief explanations of the main concepts that would be addressed in the school. They also received a reference list of papers, books, and book chapters that should be read before the Winter School started.

The 50 participants had different backgrounds (29 from physiotherapy, 18 from physical education, 2 from engineering, 1 from high school) and came from cities from the south of Brazil (border with Argentina and Uruguay) to participate in the school that was conducted based on a group-learning methodology and thinking-based learning activities. The school was advertised to other countries from Latin America and we consider that the main limitation for attendance of participants from other countries was the high cost to travel and stay abroad. All the participants had a connection with academia being either undergraduate (*n* = 24, 49%) or graduate students (*n* = 13, 26%, were master or Ph.D. students in human movement sciences or physiology), and the other 25% were young investigators with a master's or Ph.D. degree in human movement sciences or physiology, one post-doctoral fellow, and one high school student. We did not collect data regarding personal information like the age of the participants, but informally we can report that most of the participants were young with age ranging between 17 and 30 years. A higher number of female participants was noted, which may in part relate to the fact that most of the participants were from the physiotherapy field, which in Brazil usually has a higher percentage of female graduates.

Upon registration, considering a scale from zero (meaning no knowledge on signal processing) to 10 (proficient in signal analysis), only 10.2% of the registered participants indicated a score higher than 5. The school's program was designed to include theoretical and practical activities in a friendly environment. We organized a single room meeting, with coffee and finger food; lectures were delivered using media projectors, whiteboards, and questions were allowed at any time. The organizers worked to have the entire class actively participate, and when the participants showed signals of tiredness or if they missed the concepts (for example, sleepy faces, side conversations), a short break was proposed.

The main goal of the school was to promote a solid basic background, and therefore, concepts related to muscle contraction, joint angles, and ground reaction forces were briefly addressed to ensure that all participants were familiarized with the origins of biological signals that were used for the examples. In this regard, differences between kinetic, kinematic, and sEMG signals were discussed, and sEMG generation and interpretation were often mentioned, commented on, and explained during the course. Topics were organized to promote a progressive level of complexity. The entire program was covered in 3 days within a total of approximately 20 h of activities. Topics were organized in three blocks:

- 10%: the importance of the proper knowledge related to signal processing, its application in sEMG analysis, and where to find relevant material to study;
- 10%: important aspects of data acquisition, mostly related to hardware characteristics;
- 5%: examples of signal processing methods that are common among different techniques (like kinematics and kinetics) in human movements sciences;
- 75%: data processing concepts: limits, derivation, integration, sums, complex numbers, functions, Euler identity, definitions and classification of signals, discrete acquisition, Nyquist theorem, aliasing, time domain and time-series, frequency domain, Newton Prism experiment and frequency decomposition, Fourier series, harmonics, Fourier transform, spectrum, inverse Fourier, and signal filtering. We also focused on the interpretation of sEMG alterations and distortions, cross-talks, amplitude analysis, windowing functions, cancelation of signals, onset-offset analysis, frequency analysis, co-activation, synergy, and linear decomposition into basis functions. We also included some concepts of the Teager energy operator and prosthesis control, but these last topics were only briefly mentioned.

The approach to these topics was always based on evidence from the literature. Basic topics related to the mathematics involved in sEMG processing and analyses were discussed based on scientific papers, and the explanation of concepts for signal processing and analysis was always followed up by a discussion of topics related to data processing and the interpretation of results from scientific papers, i.e., topics for non-engineers began with basic math and sinusoidal wave analysis. When a practical situation was needed to illustrate concepts, as well as to discuss how a decision on data analysis or processing affects the results of sEMG analysis, scientific articles were used, i.e., filter coefficients, signal decomposition, or alias signal (aliasing). Papers considered for the examples were always related to the general study area of the participants such as those addressing gait biomechanics, jump landing, and for some cases, papers reporting the use of EMG associated with prosthesis control and biofeedback, especially to give a contextualized health scenario for non-engineers.

The voluntary tutors to the school were one professor with a Ph.D. in human movement sciences, two Ph.D. students in biological sciences, and one MSc. in engineering, and a MSc. in kinesiology and clinical biomechanics. In addition to the handout and the introductory references already mentioned, the teaching activities were mostly based on the textbooks “Digital Signal Treatment” (25), “Discrete-time Signals Processing” (26), “Electromyography: Physiology, Engineering and Non-invasive Applications” (10), “Biomechanics and Motor Control of Human Movement” (27), and the SENIAM guidelines (28, 29). The tutors worked together in the months preceding the school to prepare the material considering similar terminology, to connect the examples used, and considering common references. In the different activities of the school, signal-processing concepts were represented using Matlab 2016a (MathWorks, Massachusetts, USA) and a custom-made code shared with the Winter School participants (see Supplemental file). This environment was used to facilitate the tutors' enrollment since they all had experience with this tool, but during the school, other tools, including open-source and free options like R studio (RStudio, Inc., Boston, United States) and Python (Python Software Foundation, Wilmington, United States) were frequently commented on.

An important concern when planning the school was the need for a follow up on the educational activities, and the importance of revisiting the concepts discussed during the lectures. This is why all the lectures were video recorded and uploaded with free access on YouTube®. Lectures are available for free in the non-monetized official channel of the Applied Neuromechanics Group, the NeuromechTV (http://youtube.com/neuromechTV). Slides used to explain the mathematical concepts and examples of data analyses were also uploaded to Researchgate as “*Escola de Inverno: Fundamentos de processamento de sinais discretos em biomecânica*.”

To provide us with an idea of how the school helped the participants to better understand sEMG, at the end of the Winter School, all participants underwent one written exam and filled in a survey. The exam included 15 questions on topics addressed during the lectures. The participants had 10 days to send the answers by email after the school was completed. We also requested that participants answer a short survey to provide us with a general assessment of the school. The school was funded by the Universidade Federal do Pampa, the Brazilian Society of Biomechanics, and the Brazilian Physiology Society, without registration fees for participation.

## Main Outcomes and Discussion

The general evaluation of the school involved rating the activities from 0 (zero, meaning low quality) to 10 (ten, high quality). Participants graded the advertisement given to the school when registration was open as 9.83. The handout and reference list provided upon registration were considered pertinent, and about 50% of the participants said they read the material before the school started. The strategies employed to discuss the concepts on signal processing were graded as 9.33, and the performance of the tutors was graded as 8.83. The overall grade for the activities was 9.85, and the free registration was considered to be of 60% importance on a scale from 0 to 100, in which we asked whether participant registration was conditioned by the free registration.

The written exam completed by participants after the school showed average effectiveness of 71 ± 20% (ranging from 40 to 100%) in properly answering the 15 questions related to the topics studied. We observed that better answers were found for questions addressing those topics in which more time was spent on during the lectures and for topics we highlighted as important during the lectures (filtering and data visualization, for example).

The school had a significant number of undergraduate students from physical education and physiotherapy courses who participated, as we detailed in the previous section. As observed, when experiments and data processing and analysis using electrocorticography techniques were developed with undergraduate students, the enrollment in educational activities similar to that developed in our school may help increase their interest in following a postgraduate science program (30). Furthermore, students of different levels were developing activities together in the school. We consider that the merging of students from different levels can benefit those facing difficulties with the contents in laboratory calculations (24).

We do not believe that the different backgrounds of the participants limited the development of the activities in the school. Nevertheless, in schools aimed at more complex topics, or those prepared to solve particular problems, the heterogeneity of the participants may have different effects on the activity outcomes. We did not strictly control the methods of each activity, but in general, we conducted a team-based learning approach, which is also known to benefit learning in the laboratory environment (31). We also consider that the model of collaboration between students during the course has been beneficial for those students at a higher level of knowledge, as the teaching process increases memory acquisition and persistence in the context of medical sciences, a background from which most of the participants originated (32). We also consider that the interaction between the students from different levels can be a contributing factor to promote networks and mutual support, which in the end will help to reduce barriers in the use of sEMG. Informal conversations during the course also helped to bring students and speakers together.

Considering the topics addressed in the school, a good amount of time was spent on explaining filtering, especially because filtering is a fundamental step in the sEMG data analysis because of its susceptibility to low-frequency noise, baseline noise, and movement artifact noise (10, 27). Filtering was discussed considering pieces of evidence from the literature concerning the most adequate cut off frequencies, filter design, and the criteria for its determination (33). With this approach, we also wanted to reduce the barriers that students face when reading scientific papers and not properly understanding why and how the sEMG signals were processed. In addition to the temporal series analysis, which we considered as the most important when reading papers, we also discussed frequency analysis. Different examples were used to illustrate the applications of frequency analysis, including its potential to detect noise and to identify patterns of groups/clusters that may also help to identify movement deficiencies, for example (34).

The activities developed in the school also considered concepts related to data acquisition. We aimed to provide basic concepts and straightforward recommendations on how a sEMG examination should be conducted. When it comes to professor basic knowledge of sEMG procedures is required, improving teaching practices as well, and we consider that a specific school for teachers would be a valuable activity for the future. The results from sEMG examination can be a good platform to promote learning sessions and tutorials on how muscles are controlled, to discuss concepts of supraspinal control, reflexes, movement production, and regulation (35). Although it was not the main topic of the school, we provided basic concepts and discussions on muscle-computer interfaces and the use of sEMG for controlling external devices. This topic was addressed to stimulate the students to generate innovation by showing possibilities and enriching their experience (36). The program included a frequent revision of the basic concepts and more applied examples were discussed. We judge that this strategy was satisfactory in preventing some of the students from getting lost in the information flow. It became clear during the school that in addition to the basic physiological and mathematics concepts, there are technical aspects of sEMG that can also be considered a barrier to the correct methodological use of sEMG in daily clinical practice. These mixed limitations between math and physiology are frequently observed for health students and professionals. For example, in Chile, most students using sEMG never integrate math and physiology knowledge, and many professionals use the sEMG in clinical practice in a qualitative manner to justify rehabilitation decisions but with weak scientific support i.e., some private hospitals or universities have sEMG' devices but as the use of this devices in the analysis of treatment require time and knowledge, devices are not used well. Adequate data acquisition or analyses are frequently negatively affected by the economic pressure to treat the largest number of patients possible in minimal time. It also occurs with academic professors, who frequently have to lecture a higher number of hours in precarious conditions without time to properly address important technical aspects of sEMG. This is similar to many realities in Brazil and other countries in Latin America as well. As previously discussed, this condition affects different areas of education (6). Therefore, we consider that our approach helped to reduce this barrier as well.

We used a strategy of continuous online education by making all the lectures of the school permanently available online after the school was completed. Webinars and online classes are not a novelty these days, but most of the crash courses available are segmented for a very specialized audience, and sometimes assume that the viewers already have a good background. We believe that the recordings available online could promote additional learning when participants watch the classes again, also resulting in revalidation of the concepts, similar to what has been discussed when records of surgical procedures are integrated into patient care (37). Furthermore, the online lectures may serve as an introductory course to signal processing applied to human movement sciences, especially sEMG. The decision to make the lectures available online on YouTube® was because YouTube® was reported by 76% of physiology students as a primary source of learning (38). In the same study, 94% of students indicated that they would first search for answers online if they did not understand something in physiology, but only 31% check the sources (38). More importantly, online lectures may help other students and professionals who were not able to attend the school to learn from the lectures and to reduce barriers faced when using sEMG. While the population was struggling with the SARS-COV-2 pandemic in 2020, digital teaching and live meetings have confirmed the relevance and the utility of this strategy, benefiting learning in health and human movement sciences.

Finally, the main barriers identified by the tutors and participants were mathematical skills or background, i.e., algebra, calculus, and mathematical logical thinking. Furthermore, in many cases, fear of the numbers and programming a computer, and the insecurities and anxiety about colleagues from developed countries—who might be better prepared—were other barriers identified among the participants in Latin America. However, important social barriers also exist in regions where investment in research is not aligned with the economical aims of institutions, thus negatively impacting science education. We therefore recommend the organization of basic science schools for health careers in Latin-America, and to frequently develop critical lectures to fill educational gaps, using theoretical frameworks similar to those of engineering and other sciences, but adding innovative learning strategies from modern pedagogy in health sciences, that considers the biological profile of the students and professionals of health and life sciences.

## Conclusion

The Winter School had a good impact on participant's formation and may have motivated other groups from Brazil to promote similar educational activities during 2019 as we observed schools in topics related to biomechanics and muscle function being promoted. From the participant's point of view, the activity was excellent, and the follow up of the school on the YouTube® channel, considering the social media engagement, suggests that combining face to face activities followed by online availability of lectures can be a valid strategy to sustain the interest of students on these topics.

We recommended frequent organizations of basic science Schools in health careers in Latin-America at all levels i.e., bachelor, Master, PhD, and post-doc, as an initiative to fill educational gaps using theoretical frameworks similar to those that engineering and other sciences adopt, as innovative learning strategies for modern pedagogy in health sciences is advisable. Furthermore, we believe that providing schools that are conducted 100% online could significantly benefit a larger number of students and professionals.

## Ethics Statement

Ethical review and approval was not required for the study on human participants in accordance with the local legislation and institutional requirements. Written informed consent to participate in this study was provided by the participants.

## Author Contributions

CD, AM, and MK: conceived the project; delivered lectures; analyzed data; wrote and approved the final version of the manuscript. FC: conceived the project; approved funding; delivered lectures; analyzed data; wrote and approved the final version of the manuscript. All authors contributed to the article and approved the submitted version.

## Conflict of Interest

The authors declare that the research was conducted in the absence of any commercial or financial relationships that could be construed as a potential conflict of interest.
