# Supplementary material for: Winter School on sEMG Signal Processing: An Initiative to Reduce Educational Gaps and to Promote the Engagement of Physiotherapists and Movement Scientists With Science
Source: Front Neurol. 2020 Jun 24;11:509. doi: 10.3389/fneur.2020.00509 (PMC7326787; doi:10.3389/fneur.2020.00509)
Supplement: Supplementary file 1 [file Table_1.DOCX]

% Escola de processamento do sinais sEMG 2018

% UNIPAMPA

% Carlos De la Fuente.^(1,2,3), Alvaro S Machado.^(4), Marcos R Kunzler.^(4), Felipe P Carpes.^(4)

%

% 1 Carrera de Kinesiologia, Departamento de Cs. De la Salud, Facultad de Medicina, Pontificia Universidad Catolica de Chile, Santiago, Chile.

% 2 Laboratorio LIBFE, Escuela de Kinesiologia, Universidad de los Andes, Santiago, Chile.

% 3 Centro de Salud Deportivo, Clinica Santa Maria, Santiago, Chile.

% 4 Laboratory of Neuromechanics, Universidade Federal do Pampa, Uruguaiana, Brazil.

%

% Instructions:

% run by sections

% Create a folder and add the data file named 'emg.csv'

%% 1) sinal digital vs analogico

clear all; clc

a=1; fs=1; t=linspace(0,360,360); phase=0;

A = a*sin(2*pi*fs*t+phase);

a2=1; fs2=1; t2=linspace(0,10,10); phase2=0;

B = a2*sin(2*pi*fs2*t2+phase2);

figure(1)

plot(t,A); title(strcat('seno', '', num2str(fs),'' ,'Hz')); ylabel('Intensidad (a.u.)'); xlabel('angulo(o)')

ylim([-max(A)*1.2 max(A)*1.2]); xlim([0 size(A,2)]);

hold on; stem(linspace(0,360,10),B); title(strcat('seno', '', num2str(fs),'' ,'Hz')); ylabel('Intensidad (a.u.)'); xlabel('angulo(o)')

ylim([-max(A)*1.2 max(A)*1.2]); xlim([0 size(A,2)]);

legend('Analog','digital')

%saveas(figure(2),'figura2.jpg')

%% 2) resumo sinal digital vs analogico

n=0;

for i=0.1:2:30

n=n+1;

y(:,n) = awgn(A,i)'; % toolbox of comunication system

end

signal=y(:,8); %plot(signal)

%ruido=signal-A;

%filtrar

[c,d]=butter(2,1/(100/2),'low');

for j=2:size(signal,1)-1

signal_t=filtfilt(c,d,signal);

end

clear ('i','j','c','d')

figure(2)

plot(signal_t)

title('filtered')

%% SnR multiples

figure(4)

subplot (5,2,1), plot(y(:,1));xlim([0 360]);title('Sinais com ruido');subplot (5,2,2), histogram(y(:,1));xlim([-4 4]);title('histograma do sinal')

subplot (5,2,3), plot(y(:,4));xlim([0 360]);subplot (5,2,4), histogram(y(:,4));xlim([-4 4])

subplot (5,2,5), plot(y(:,8));xlim([0 360]);subplot (5,2,6), histogram(y(:,8));xlim([-4 4])

subplot (5,2,7), plot(y(:,12));xlim([0 360]);subplot (5,2,8), histogram(y(:,12));xlim([-4 4])

subplot (5,2,9), plot(y(:,15));xlim([0 360]);subplot (5,2,10), histogram(y(:,15));xlim([-4 4])

%% Fourier

C=1*cos(2*pi*(100*2)*linspace(0,50,1000));

D=1*cos(2*pi*(140*2)*linspace(0,50,1000));

E=1*cos(2*pi*(80*2)*linspace(0,50,1000));

F=C+D+E;

figure(5);subplot(2,1,1), plot(C); hold on; plot(D), plot(E); legend('2*pi*(100*2)','2*pi*(140*2)','2*pi*(80*2)','Location','northoutside','Orientation','horizontal')

subplot(2,1,2), plot(F); legend(' sum of cos(2*pi*(100*2) + cos(2*pi*(140*2)) + cos(2*pi*(80*2))','Location','northoutside','Orientation','horizontal')

F=F';C=C';D=D';E=E'; Fs=280;

sig=F;

NFFT = 2^nextpow2(numel(sig)); % potencia de 2 seguinte a numel(data(:,2))->una emg

Y= fft(sig,NFFT)/numel(sig); % Algoritmo Fast fourier transform

f = Fs/2*linspace(0,1,NFFT/2+1); % frequencia de amostragem de acordo com Nyquist + vetor horizontal

X = 2*abs(Y(1:NFFT/2+1));

figure(6); plot (f,2*X); ylabel('FFT(Y)'); xlabel('Frequency(Hz)'); title('Real part: sum of cos(2*pi*(100*2) + cos(2*pi*(140*2)) + cos(2*pi*(80*2))')

%% filter butterworth low, high,

%[b,a] = butter(3,[0.2 0.6],'stop'); dataOut = filter(b,a,dataIn);

y1=y(:,5); n=6

for i=0.05:0.05:0.49

n=n+1

figure(n); plot(y1)

[c,d]=butter(1,i/(1/2),'low'); % Guzman-venegas et al. PLoS One. 2015;10(2):e0116923.

y_fil=filtfilt(c,d,y1);

hold on; plot(y_fil)

title(strcat('butter(1,i/(1/2),low), i =',num2str(i)));legend('Raw','filtered')

end

%close all

%% EMG: importa??o do sinal

clear all; clc; close all;

[filename,path] = uigetfile('*.csv','Select the file with the signals','MultiSelect','on');

cd(path)

Data1= importdata(filename);

%% show signal

tempo=Data1(:,1);

signal= Data1(:,2);

Fs=1000

NFFT = 2^nextpow2(numel(signal)); % potencia de 2 seguinte a numel(data(:,2))->una emg

Y= fft(signal,NFFT)/numel(signal); % Algoritmo Fast fourier transform

f = Fs/2*linspace(0,1,NFFT/2+1); % frequencia de amostragem de acordo com Nyquist + vetor horizontal

X = 2*abs(Y(1:NFFT/2+1));

figure(1);

subplot(1,2,1),plot(tempo,signal); title('Dominio do tempo'); ylabel('Voltagem(V)'); xlabel('Tempo(s)');xlim([0 max(tempo)])

subplot(1,2,2),plot (f,2*X); ylabel('FFT(Y)'); xlabel('Frequency(Hz)');title('Dominio da frequencia')

%% OFFset problem

figure(2);

plot(tempo,signal+0.5)

hold on; plot(tempo, linspace(0,0,size(signal,1)),'k-'); title('Offset problems')

legend('EMG signal','Zero-line')

%% AC problem

signal= Data1(:,10);

AC=0.15*sin(2*pi*50*linspace(0,max(tempo),size(signal,1)))';

figure(3);plot(tempo,AC); title('AC'); ylim([-1 1])

signal=signal+AC;

NFFT = 2^nextpow2(numel(signal)); % potencia de 2 seguente a numel(data(:,2))->una emg

Y= fft(signal,NFFT)/numel(signal); % Algoritmo Fast fourier transform

f = Fs/2*linspace(0,1,NFFT/2+1); % frequencia de amostragem de acordo com Nyquist + vetor horizontal

X = 2*abs(Y(1:NFFT/2+1));

figure(4);

subplot(1,2,1),plot(tempo,signal); title('Dominio do tempo'); ylabel('Voltagem(V)'); xlabel('Tempo(s)');xlim([0 max(tempo)])

subplot(1,2,2),plot (f,2*X); ylabel('FFT(Y)'); xlabel('Frequency(Hz)');title('Dominio da frequencia')

%% Movemento

mov=0.03*sin(2*pi*2*linspace(0,max(tempo),size(signal,1)))';

signal=signal+mov;

NFFT = 2^nextpow2(numel(signal)); % potencia de 2 seguente a numel(data(:,2))->una emg

Y= fft(signal,NFFT)/numel(signal); % Algoritmo Fast fourier transform

f = Fs/2*linspace(0,1,NFFT/2+1); % frequencia de amostragem de acordo com Nyquist + vetor horizontal

X = 2*abs(Y(1:NFFT/2+1));

figure(5);

subplot(1,2,1),plot(tempo,signal); title('Dominio do tempo'); ylabel('Voltagem(V)'); xlabel('Tempo(s)');xlim([0 max(tempo)])

subplot(1,2,2),plot (f,2*X); ylabel('FFT(Y)'); xlabel('Frequency(Hz)');title('Dominio da frequencia')

%% offset (centering signal)

signal= Data1(:,10);

figure(6);

subplot(1,2,1), plot(tempo,signal+0.5); title('Offset ')

subplot(1,2,2), plot(tempo,signal-mean(signal)); title('Sem Offset')

%% Pass-band

cutoff1=450

cutoff2=20

figure(6);

subplot(1,3,1), plot(tempo,signal); title('sem passabanda '); ylim([-5 5])

subplot(1,3,3), plot(tempo,signal);

[c,d]=butter(2,cutoff1/(Fs/2),'low'); % Guzman-venegas et al. PLoS One. 2015;10(2):e0116923.

signal=filtfilt(c,d,signal);

[a,b]=butter(2,cutoff2/(Fs/2),'high');

signal=filtfilt(a,b,signal);

figure(6);

subplot(1,3,2), plot(tempo,signal); title('com passabanda '); ylim([-5 5])

subplot(1,3,3), hold on; plot(tempo,signal);title('[2 400]'); ylim([-5 5])

%% reject band

signal= Data1(:,10);

AC=0.15*sin(2*pi*50*linspace(0,max(tempo),size(signal,1)))';

cutoff1=60

cutoff2=40

figure(7);

subplot(1,2,1), plot(tempo,signal); title('com filtro'); ylim([-5 5])

[b,a] = butter(3,[cutoff2/(Fs/2) cutoff1/(Fs/2)],'stop');

signal=filtfilt(b,a,signal);

NFFT = 2^nextpow2(numel(signal)); % potencia de 2 seguinte a numel(data(:,2))->una emg

Y= fft(signal,NFFT)/numel(signal); % Algoritmo Fast fourier transform iterado

f = Fs/2*linspace(0,1,NFFT/2+1); % frequencia de amostragem de acordo com Nyquist + vetor horizontal

X = 2*abs(Y(1:NFFT/2+1));

figure(7); subplot(1,2,2), plot (f,2*X); ylabel('FFT(Y)'); xlabel('Frenquency(Hz)');title('Dominio da frequencia')

signal_rec= ifft(Y,size(signal,1),'symmetric');

figure(8);

subplot(1,2,1), plot(tempo,signal_rec);

subplot(1,2,2), plot(tempo,signal);

%% Rectification

signal= Data1(:,10);

signal_2=signal*0;

for i=1:size(signal,1)

if signal(i,:)>0

signal_2(i,:)=signal(i,:);

end

end

signal= abs(signal);

NFFT = 2^nextpow2(numel(signal)); % potencia de 2 seguinte a numel(data(:,2))->una emg

Y= fft(signal,NFFT)/numel(signal); % Algoritmo Fast fourier transform

f = Fs/2*linspace(0,1,NFFT/2+1); % frequencia de amostragem de acordo com nyquist + vetor horizontal

X = 2*abs(Y(1:NFFT/2+1));

NFFT2 = 2^nextpow2(numel(signal_2)); % potencia de 2 seguinte a numel(data(:,2))->una emg

Y2= fft(signal_2,NFFT)/numel(signal_2); % Algoritmo Fast fourier transform

f2 = Fs/2*linspace(0,1,NFFT/2+1); % frequencia de amostragem de acordo com nyquist + vetor horizontal

X2 = 2*abs(Y(1:NFFT/2+1));

figure(9)

subplot(2,2,1), plot(signal)

subplot(2,2,3), plot (f,2*X);xlim([0.5 f(1,end)])

subplot(2,2,2),plot(signal_2)

subplot(2,2,4), plot (f2,2*X2);xlim([0.5 f2(1,end)])

%% Windowing

clear('Data_rms')

window_ms=500

porcentagem=0

paso_ms=fix(window_ms*((100-porcentagem)/100));

windows_pts=fix(window_ms*10^-3*Fs)

paso=fix(paso_ms*10^-3*Fs)

k=1;

for j=1:paso:size(signal,1)-windows_pts

k=k+1;

Data_rms(k,:)= rms(signal(j:j+windows_pts-1,:));

end

overlap=(windows_pts-paso)*100/windows_pts % Se for 0% ? overlap, se ? negativo tem separa??o com overlap zero, se ? positivo tem overlap das janelas.

plot(Data_rms); title(strcat('Janela',num2str(window_ms),'ms, overlap',num2str(porcentagem),'%'))
